# Nanoscale surface chemistry directs the tunable assembly of silver octahedra into three two-dimensional plasmonic superlattices

**DOI:** 10.1038/ncomms7990

**Published:** 2015-04-29

**Authors:** Yih Hong Lee, Wenxiong Shi, Hiang Kwee Lee, Ruibin Jiang, In Yee Phang, Yan Cui, Lucio Isa, Yijie Yang, Jianfang Wang, Shuzhou Li, Xing Yi Ling

**Affiliations:** 1 Division of Chemistry and Biological Chemistry, School of Physical and Mathematical Sciences, Nanyang Technological University, Singapore 637371, Singapore; 2 Division of Materials Science, School of Materials Science and Engineering, Nanyang Technological University, Singapore 639798, Singapore; 3 Institute of Materials Research and Engineering, A*STAR (Agency for Science, Technology and Research), 3 Research Link, Singapore 117602, Singapore; 4 Department of Physics, The Chinese University of Hong Kong, Shatin, Hong Kong, China; 5 Laboratory for Interfaces, Soft matter and Assembly, Department of Materials, ETH Zurich, Vladimir-Prelog-Weg 5, Zurich 8093, Switzerland

## Abstract

A major challenge in nanoparticle self-assembly is programming the large-area organization of a single type of anisotropic nanoparticle into distinct superlattices with tunable packing efficiencies. Here we utilize nanoscale surface chemistry to direct the self-assembly of silver octahedra into three distinct two-dimensional plasmonic superlattices at a liquid/liquid interface. Systematically tuning the surface wettability of silver octahedra leads to a continuous superlattice structural evolution, from close-packed to progressively open structures. Notably, silver octahedra standing on vertices arranged in a square lattice is observed using hydrophobic particles. Simulations reveal that this structural evolution arises from competing interfacial forces between the particles and both liquid phases. Structure-to-function characterizations reveal that the standing octahedra array generates plasmonic ‘hotstrips', leading to nearly 10-fold more efficient surface-enhanced Raman scattering compared with the other more densely packed configurations. The ability to assemble these superlattices on the wafer scale over various platforms further widens their potential applications.

## Abstract (web summary)

The self-assembly of anisotropic nanoparticles into large-area superlattices remains challenging. Here the authors exploit surface chemistry to tune the wettability of silver nano-octahedra, and direct a continuous superlattice structural evolution, from close-packed to progressively open structures.

## Graphical abstract

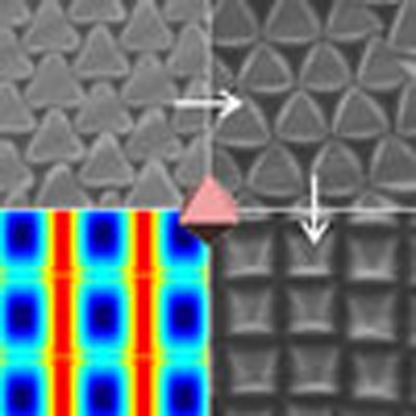

Controlled organization of nanoparticles into scalable superlattices with tunable crystal structure and spacing is a grand challenge in nanotechnology^1^. Nanoparticle superlattices exhibit unique crystal structure-dependent physicochemical functionalities, playing a pivotal role in the design of next-generation devices^2^,^3^,^4^,^5^,^6^. Several methods are commonly used to form superlattices, including varying interparticle forces among spherical particles^7^,^8^,^9^,^10^,^11^,^12^,^13^, using anisotropic nanoparticles with various morphologies^14^,^15^,^16^,^17^,^18^,^19^,^20^,^21^,^22^,^23^,^24^,^25^,^26^,^27^, and forming localized chemical patchiness on anisotropic nanoparticles^28^. However, it remains difficult to assemble multiple superlattices over large areas using just one nanoparticle morphology. Overcoming this limitation will enhance superlattice diversity, enable direct investigations of structure-to-function variations arising solely from superlattice structural changes and allow more specific customization of superlattices for targeted applications^29^. In particular, anisotropic nanoparticles possessing well-defined facets are appealing because these facets can interact with each other in various ways to create distinct superlattices^30^. However, such interactions among one type of particle morphology have not yet been extensively explored to achieve tunable self-assembled nanoparticle superlattices, particularly in the formation of non-close-packed superlattices.

Here we demonstrate the concept of ‘one anisotropic particle, multiple superlattices' using octahedral silver (Ag) nanoparticles. We are able to tailor the surface chemistry of Ag octahedra to assemble three wafer-scale two-dimensional (2D) plasmonic superlattices at the oil/water interface, one of which is a square superlattice with Ag octahedra standing on their vertices. Through molecular dynamics (MD) simulations, we find that these bespoke superlattices arise from a competition of interactions between the nanoparticles and both liquid phases. Despite having the lowest-packing density, the square superlattice is the most efficient surface-enhanced Raman scattering (SERS) superlattice. This finding demonstrates the importance of nanoscale superlattice design, where more nanoparticles do not always generate stronger scattering signals^31^, contrary to the rule of thumb in SERS research.

## Results

### Assembly of three plasmonic superlattices with one particle

**Figure 1:**
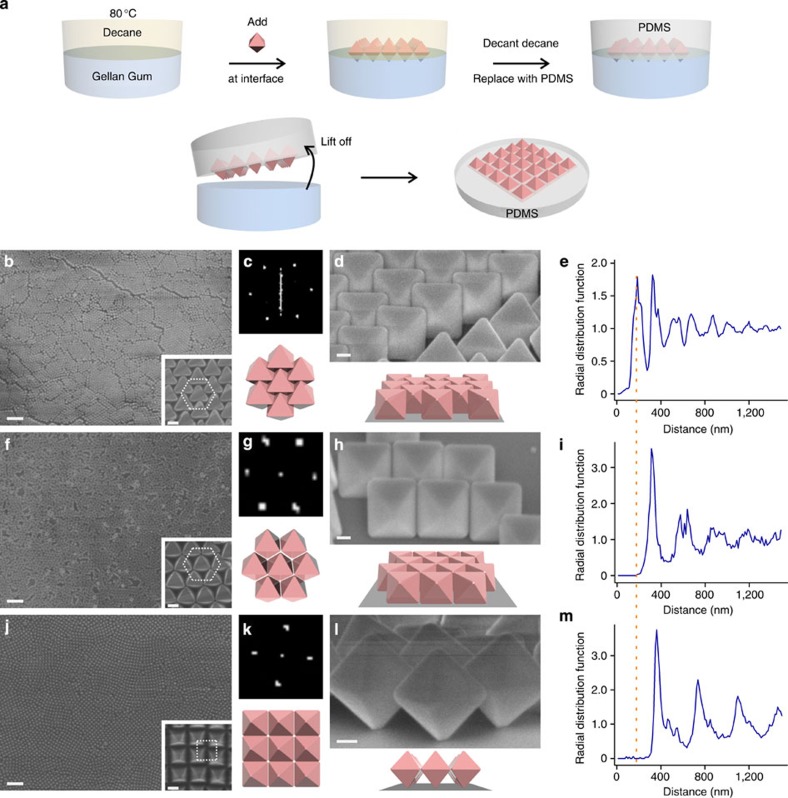
Controlling nanoscale surface wettability of Ag octahedra to assemble three superlattices. (**a**) Schematic of interfacial self-assembly protocol at the oil/water interface. (**b**–**d**,**f**–**h**,**j**–**l**) SEM characterization and corresponding fast-Fourier transforms (FFTs) of the various Ag octahedra superlattices. (**b**) HCP monolayer of PVP octahedra is confirmed with FFT (**c**), adopting a planar configuration (**d**). (**f**,**g**) The planar OH superlattice is formed using C3-octahedra, with the octahedra aligned edge to edge (**h**). (**j**,**k**) A square superlattice with a pyramidal appearance is observed using C16-octahedra. The octahedra are aligned edge to edge. (**l**) Cross-sectional SEM shows only the vertices of Ag octahedra contacting the substrate. (**b**,**f**,**j**) Scale bars, 2μm; (**b**,**f**,**j**) inset scale bars, 200nm; (**d**,**h**,**l**) scale bar, 100nm. (**e**–**m**) Radial distribution functions of the three superlattices highlight excellent long-range order. The nearest neighbour distance increases from 188 nm (**e**) to 313 nm (**i**) and to 362 nm (**m**) for the HCP, OH and square superlattices, respectively.

We employ a chemical approach to tune the surface hydrophobicity of Ag octahedra building blocks. As-synthesized Ag octahedra (edge lengths ∼356±10 nm) are stabilized with the hydrophilic polymer poly(vinylpyrrolidone) (PVP). The hydrophobicities of these particles are homogeneously increased via ligand exchange with alkylthiols of increasing chain lengths, including 1-propanethiol (C3SH) and 1-hexadecanethiol (C16SH). These Ag octahedra are then assembled at the oil/water interface and subsequently immobilized using the gel-trapping technique^32^ (Fig. 1a; Supplementary Fig. 1; Methods). Once trapped at the interface, the superlattices can be transferred to various solid platforms.

A hexagonal close-packed (HCP) 2D superlattice forms at the oil/water interface using PVP-functionalized Ag octahedra (PVP octahedra, bulk water contact angle (CA) measured on Ag film functionalized with PVP ∼(39±5)°; Fig. 1b,c). Neighbouring Ag octahedra contact via a face-to-face configuration (Fig. 1d; Supplementary Fig. 2). This superlattice is planar, with one facet of the octahedra in direct contact with the substrate (Fig. 1d). Long-range order of this superlattice is evident from the regularly oscillating curves in its corresponding radial distribution function (Fig. 1e) and honeycomb network of Voronoi cells (Supplementary Fig. 3a); the nearest neighbour distance is estimated to be 188 nm (Fig. 1e). The packing efficiency of this close-packed array is 88.9% (Supplementary Fig. 4).

The HCP superlattice changes to an open hexagonal (OH) array using C3SH-functionalized Ag octahedra for the self-assembly (C3-octahedra, CA=(83±2)°; Fig. 1f,g). A comparison of this monolayer (Fig. 1h) with the HCP array (Fig. 1d) clearly illustrates the change from a face-to-face contact to an edge-to-edge alignment. Order is also evident from the radial distribution function and Voronoi cells of this 2D array (Fig. 1i; Supplementary Fig. 3b), with the nearest neighbour distance increased to 313 nm. The packing efficiency of this monolayer decreases to 66.7% (Supplementary Fig. 5).

An entirely different square superlattice is formed by assembling hydrophobic C16SH-functionalized Ag octahedra (C16-octahedra, CA=(110±2)^o^; Fig. 1j,k). Partial submersion of edge-to-edge aligned Ag octahedra within the poly(dimethylsiloxane) (PDMS) mold gives a pyramidal appearance (Fig. 1j; inset). Cross-sectional scanning electron microscopy (SEM) image of the superlattice transferred to a Si substrate definitively shows only a single vertex of the octahedra contacting the substrate (Fig. 1l). This superlattice of standing octahedra has the lowest-packing efficiency among the three arrays at 33.3% (Supplementary Fig. 6). Superlattice crystallinity is evident from the radial distribution function (Fig. 1m) and the square Voronoi cells (Supplementary Fig. 3c), with the nearest neighbour distance estimated to be 362 nm. Notably, the three different superlattices observed heretofore arise solely from variations of Ag octahedra surface wettability.

### Interfacial behaviour of Ag octahedra

**Figure 2:**
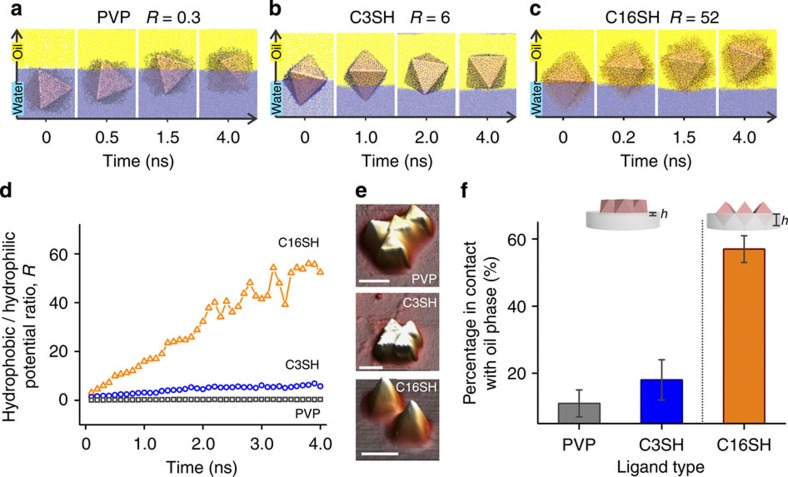
Using molecular dynamics simulations to unravel the interfacial behaviours of Ag octahedra. (**a**–**c**) Evolution of the interfacial position and configuration of Ag octahedra over simulation times of 4 ns, during which thermodynamic equilibrium is achieved. Both PVP octahedron (**a**) and C3-octahedron (**b**) attain a stable planar configuration over time. However, PVP octahedron remains mostly in contact with the aqueous phase, whereas C3-octahedron moves into the oil phase. (**c**) C16-octahedron moves across the oil/water interface, maintaining a stable standing configuration. (**d**) Plot of hydrophobic/hydrophilic potential energy ratios over time. Hydrophobic interactions become dominant as the ligands change from PVP to C16SH. (**e**) AFM topological characterization of the variously functionalized Ag octahedra on PDMS. All scale bars, 500nm. (**f**) From AFM measurements, the height percentage of the Ag octahedra in contact with the oil phase can be derived (Supplementary Fig. 8). Increasing surface hydrophobicity of Ag octahedra increases the height percentage in contact with the oil phase in the experiments. Error bars are s.d. collected from sampling at least 50 particles.

MD simulations show that Ag octahedra move spontaneously to the interface to attain thermodynamic equilibrium^33^ (Supplementary Notes 1 and 2; Supplementary Movies 1–3; Supplementary Fig. 7). PVP octahedron rotates from a tilted configuration in the aqueous phase to become planar on breaching the oil/water interface (Fig. 2a). For C3-octahedron, the particle rotates from a nearly upright configuration to attain a stable planar equilibrium configuration. In contrast, C16-octahedron adopts an upright configuration from the beginning and this configuration is maintained at equilibrium. Notably, the interfacial configurations of these Ag octahedra match well with the experimental topological characterization using atomic force microscopy (AFM; Fig. 2e). The experimental height percentages of the particle in contact with the oil phase are 11%, 18% and 57% for PVP, C3- and C16-octahedra, respectively (Fig. 2f; Supplementary Fig. 8; Supplementary Table 1). These values are different from those observed in the simulations (Supplementary Fig. 9), likely arising from different magnitudes of capillary forces due to the smaller particle sizes used in the simulations^33^.

The interfacial behaviour of Ag octahedra is dictated by the competition between the particle–water and particle–oil interactions, defined as the hydrophobic/hydrophilic potential energy ratio. This ratio increases from 0.3 to 6, and to 52 as the ligands change from PVP to C3SH, and to C16SH, respectively (Fig. 2d; Supplementary Fig. 10). Particle–oil interaction becomes increasingly favourable with increasing hydrophobicity of the ligands. Consequently, PVP octahedron remains in contact with the aqueous phase due to a favourable ligand–water interaction, forming the HCP superlattice. C3-octahedron moves further into the oil phase with a stronger particle–oil interaction, suggesting that an OH lattice is desirable to achieve an optimal interaction with both phases. In contrast, dominant particle–oil interaction for C16-octahedron drives it into a standing configuration to minimize contact with the aqueous phase, giving rise to a square superlattice of standing octahedra aligned edge to edge.

### Tuning superlattice structure using surface hydrophobicity

**Figure 3:**
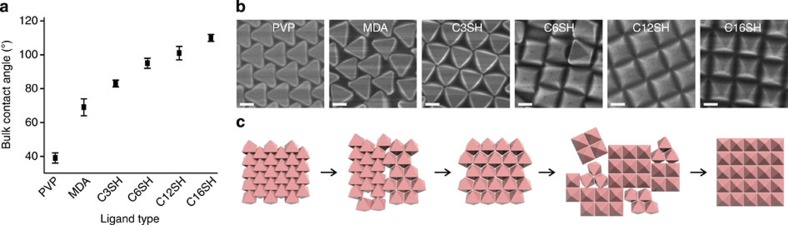
Systematic tuning of surface wettabilities creates continuous superlattice structural change. (**a**) Increase in the bulk water contact angles of a Ag film functionalized with various thiols. Error bars are s.d. of five measurements for each ligand. (**b**) Using increasingly hydrophobic Ag octahedra for self-assembly gives rise to a continuous superlattice evolution, with the HCP superlattice changing into the OH structure and finally into the square superlattice. All scale bars, 200nm. (**c**) Schematic illustration of the relationship between the three superlattices.

**Figure 4:**
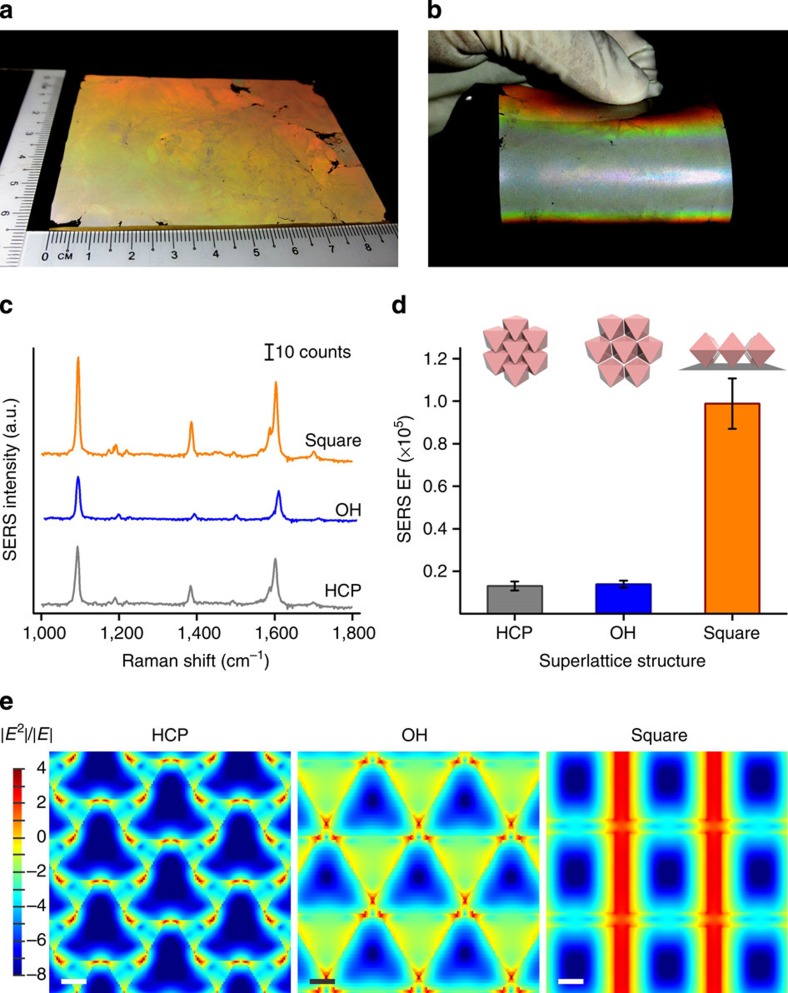
Structure-to-function optical characterization of the three 2D plasmonic superlattices. (**a**,**b**) Bending the ∼60 cm^2^ flexible PDMS film of standing octahedra superlattice (**a**) leads to unique colour changes (**b**). (**c**,**d**) SERS spectra (**c**) and EFs (**d**) of 4-MBT from the three superlattices. Error bars in (**d**) are s.d. of 10 measurements (area of each measurement is 60 × 3 μm^2^). The low-packing density square superlattice of standing octahedra is an order of magnitude more efficient than the hexagonal lattices. (**e**) Localized electromagnetic field distributions of the three superlattices. All scale bars, 100 nm. Large-area plasmonic ‘hotstrips' in the square superlattice is observed. 4-MBT, 4-methylbenzenthiol.

By systematically using Ag octahedra functionalized with different alkylthiols (to impart different hydrophobicity to the particles) for self-assembly (Fig. 3a), we show that the superlattice structures observed are related to each other (Fig. 3b; Supplementary Fig. 11). The hydrophobicity of the alkylthiols we use increases in the order of PVP<12-mercaptododecanoic acid (MDA)<C3SH<1-hexanethiol (C6SH)<1-dodecanethiol (C12SH)<C16SH (Fig. 3a). The HCP superlattice quickly loses its crystallinity as the PVP octahedra is changed to the slightly more hydrophobic MDA-octahedra (CA=(69±5)°). Changing MDA-octahedra to C3-octahedra leads to the formation of the OH array; a mixture of planar and standing octahedra clusters forms when C3-octahedra is replaced by C6-octahedra (CA=(95±3)°). The population of the standing octahedra increases markedly with the use of C12-octahedra (CA=(101±4)°). A schematic illustrating the relationship between the superlattices is shown in Fig. 3c. While the oil/water interface is quintessential for the formation of the standing octahedra superlattice (Supplementary Figs 12,13), the formation of the standing octahedra superlattice is insensitive to the locations at which the C16-octahedra are added in the presence of both phases (Supplementary Fig. 14). Our self-assembly protocol enables versatile superlattice transfer onto various polymeric platforms (Supplementary Fig. 15), Si substrates (Supplementary Fig. 16), and scaling up to ∼60 cm^2^ (Fig. 4a). Bending this large mold leads to distinct colour changes (Fig. 4b), likely arising from the angle-dependent Bragg reflection of incident light off the periodic pyramidal superlattice.

### SERS behaviours of the plasmonic superlattices

A structure-to-function characterization of the SERS capabilities of these three superlattices highlights the superior performance of the square superlattice over the hexagonally tiled ones. Constructing different superlattices without changing nanoparticle morphology allows us to single out the influence of superlattice structure on the corresponding SERS performance. Using 4-methylbenzenthiol as the probe molecule, fingerprint vibrational modes at 1,080 and 1,600 cm^−1^ are observed (Fig. 4c; Supplementary Table 2). The SERS enhancement factor for the 1,080 cm^−1^ mode of the standing array is estimated to be 9.9 × 10^4^, 7.5-fold higher than both the hexagonal arrays (Fig. 4d; Supplementary Note 3).

This difference in SERS enhancement factors of the three superlattices arises from the stark contrast in their plasmonic hot spot distribution. The local electromagnetic field distribution for the Ag octahedra superlattices along the surface of the PDMS molds is simulated using finite-difference time-domain (FDTD) method. At the incident laser excitation wavelength of 532 nm, plasmonic hot spots are highly localized in both hexagonal superlattices to regions of contacting points between neighbouring Ag octahedra (Fig. 4e). On the other hand, strips of plasmonic hot spots are formed in the standing array: hot spots spread over the entire edge length of the exposed Ag octahedra and are almost continuous along the entire array across neighbouring Ag octahedra. An estimate of the hot spot areas in these arrays indicates that a single hot spot is almost 130-fold larger in the standing array compared with the hexagonal lattices (∼22,650 versus ∼360 nm^2^). Furthermore, the pyramidal structure of the standing octahedra array enables efficient scattering of SERS signals^34^. These factors collectively lead to much stronger SERS enhancement in the square standing superlattice. Notably, this SERS study indicates that ‘more is not always better' for SERS, since the lowest-packing density superlattice is the most efficient SERS substrate. Our finding shows that structural design on the nanoscale can significantly impact the resulting macroscopic optical behaviours, owing to the nanoscale unique and structure-specific light–matter interactions. We believe this finding will inspire future work to factor in the relevance of nanoscale organization in designing functional and efficient materials.

## Discussion

In this work, we demonstrate the ability to assemble three bespoke 2D plasmonic superlattices by tailoring the surface chemistries of Ag octahedra. By controlling the hydrophobic/hydrophilic interactions of the octahedral particles at the oil/water interface, we demonstrate the relationship between the three superlattices. The homogeneity of surface functionalities also enables the superlattices to be assembled on the wafer scale, bridging the gap between nanoscopic materials and macroscopic applications. To this end, we further highlight the importance of superlattice design in tailoring nanoscale light–matter interactions for efficient macroscopic sensing applications.

## Methods

### Nanocrystal synthesis

The preparation of Ag octahedra was carried out using the polyol reduction method^35^, starting first with the synthesis of Ag nanocubes. In a typical nanocube synthesis, 10 ml of CuCl_2_ (8 mg ml^−1^), PVP (20 mg ml^−1^) and AgNO_3_ (20 mg ml^−1^) were separately dissolved in 1,5-pentanediol (PD). 35 μl CuCl_2_ solution was added to the AgNO_3_ solution. 20 ml PD was then heated to 190 °C for 10 min. 250 μl PVP precursor was added to a round-bottom flask dropwise every 30 s, while 500 μl AgNO_3_ precursor was injected into the flask every minute in one go. The reaction was allowed to proceed for ∼20 min following which, the injection was continued using 30 ml of PVP (20 mg ml^−1^) and AgNO_3_ (40 mg ml^−1^ with 120 μl CuCl_2_). Both precursors were separately prepared in PD. The reaction was allowed to proceed until the precursors were used up. The Ag octahedra solution was redispersed in 20 ml ethanol after removing the PD via multiple centrifugation rounds, and diluted, then vacuum filtered multiple times using polyvinylidene difluoride filter membranes (Durapore) with pore sizes ranging from 5,000, 650, 450 to 220 nm to remove impurities before finally dispersing in ethanol. Following purification, the PVP-functionalized Ag octahedra were subjected to ligand exchange reactions to tailor their surface chemistry for subsequent self-assembly experiments.

### Ligand exchange reactions

The purified Ag octahedra dispersion was generally allowed to sediment. Sediment (0.5 μl) was dispersed in ethanol and centrifuged once more before dispersing in 1.5 ml of ethanol/isopropyl alcohol (1:1). 100 μl of 10 mM thiol solution (MDA, C3SH, C6SH, C12SH and C16SH) was then added dropwise to this dispersion under stirring. Ligand exchange was allowed to take place for 4 h, followed by two rounds of centrifugation, redispersal in 1.5 ml of ethanol/isopropyl alcohol (1:1) and addition of fresh thiol solution under stirring. This step was allowed to continue for another 3 h, followed by three rounds of centrifugation and washing with isopropyl alcohol/water (1:1). Subjecting Ag octahedra to two rounds of ligand exchange removed the replaced PVP and facilitated subsequent ligand exchange. For ligand exchange with thermally evaporated Ag film, the substrate was immersed in a 10 mM thiol solution for at least 12 h to allow the self-assembled monolayers to form before rinsing with ethanol to remove the excess unbound thiols.

### Interfacial self-assembly and gel-trapping experiments

The gel-trapping experiments are illustrated in Fig. 1a. Gellan gum aqueous solution (2 wt%) was used as the water phase and n-decane was used as the oil phase instead. The gel-trapping experiments were slightly modified from the procedure reported by Paunov and co-workers^32^. The gellan gum solution was first heated to ∼80 °C in an oil bath to ensure that the gel was fully hydrated. Subsequently, pre-heated n-decane was added to the top of the gellan gum solution to create the oil phase. Ag octahedra were then added to the oil/water interface and the entire mixture was left at 80 °C for ∼15 min before being allowed to cool slowly to room temperature. This ensured that the nanoparticles had sufficient time to spread and achieve thermodynamic equilibrium at the oil/water interface. Once the gel had set in the aqueous phase, the oil phase was then decanted gently. In place of n-decane, a layer of premixed PDMS precursor mixture (5:1; elastomer:curing agent) was poured over the nanoparticle monolayer and the container was left at room temperature for the PDMS to cure. After the PDMS had hardened, it was lifted off from the gel and washed in hot water. For transfer to other polymeric films, 1 ml of either poly(methyl methacrylate) (PMMA)/toluene solution or poly(3-hexylthiophene-2,5-diyl) (P3HT)/toluene/chloroform was added in place of PDMS and allowed to evaporate before lift-off. For interfacial self-assembly using hexane as the oil phase, Si substrates were immersed within the water phase before the assembly process. Ag octahedra was typically dispersed in a isopropyl alcohol/water (1:1) mix and added dropwise to the interface. The Ag octahedra monolayer was allowed to stabilize for around 15 min before film transfer to the substrates. The substrates were slowly dipped up into the hexane phase using the motorized dipper of a Langmuir–Blodgett machine (KSV Nima, KN1002) at a rate of 25 μm s^−1^ for the transfer of Ag octahedra monolayer onto the substrates. After emerging from the hexane phase, the substrates were allowed to dry under ambient conditions.

### Contact angle measurements

Contact angles of the functionalized 114-nm-thick Ag films were measured on a Theta Lite tensiometer equipped with a Firewire digital camera. Static contact angle was measured with a 4-μl ultrapure sessile water droplet. A total of five readings were taken at different spots on the same substrate for each of the thiol-functionalized Ag film and averaged to obtain the bulk contact angles.

### AFM experiments

AFM was used to investigate the topological changes in the self-assembled Ag octahedra superlattices on the PDMS molds using a Bruker Dimension ICON with NanoScope V controller from Bruker. Tapping mode (non-contact mode) image was acquired using silicon probes (Tap300Al-G with 30-nm aluminium reflex coating) from BudgetSensor. Data analysis was carried out using WSxM Scanning Probe Microscopy Software, a free program from Nanotec Electrónica S.L^36^.

### Quantitative image analyses

Long-range order of the various 2D octahedra arrays was characterized using the freeware ImageJ. The radial distribution function profiles were analysed by converting the SEM images into a binary format, followed by the automatic identification of the centres of the Ag octahedra. Voronoi–Delaunay cells were constructed using custom-written IDL (Exelis) algorithms. The centre of each particle (or each facet for the hexagonal superlattices) was found by locating local intensity maxima after filtering the SEM images with a band-pass filter, followed by constructing the Voronoi–Delaunay cells.

### MD simulations

All MD simulations were conducted on single Ag octahedron placed at the oil/water interface using the GROMACS 4.07 simulation package^37^ and GROMOS96 force fields^38^. See Supplementary Fig. 17, Supplementary Note 1 and Supplementary Tables 3–5 for detailed discussion.

### SERS experiments

SERS measurements were performed using the x–y imaging mode of the Laser Raman Microscope RAMANtouch system with an excitation wavelength of 532 nm (power=3 μW). A × 100 (numerical aperture 0.9) objective lens with 5 s accumulation time was used for data collection between 200 to 1,800 cm^−1^. X–y imaging measurements were collected from 10 different spots with the area of each spot spanning 60 × 3 μm^2^. The probe molecule used was 4-methylbenzenethiol and was place exchanged onto the superlattices.

### FDTD calculations

FDTD simulations were performed using FDTD Solution 7.5, which was developed by Lumerical Solutions, Inc. The periodic boundary was used to simulate the nanostructure arrays. During the simulations, an electromagnetic pulse in the wavelength range 300−1,200 nm was launched to simulate a propagating plane wave interacting with the nanostructure. The unit cells of nanostructure arrays and its surrounding medium were divided into meshes of 0.5 nm in size. All nanostructure arrays were built according to the experimental parameters. The refractive index of PDMS was taken to be 1.4 (ref. 39) and the refractive index of air was set to be 1. The dielectric function of silver was taken from previously reported values^40^. The electric field intensity at wavelength of 532 nm was monitored at the surface of PDMS.

## Author contributions

Y.H.L. and X.Y.L. designed research; Y.H.L., H.K.L., I.Y.P., Y.C. and Y.Y.J. performed research; W.X.S. and S.Z.L. performed MD simulations; R.B.J. and J.F.W. performed FDTD simulations; L.I. performed image analyses; Y.H.L., W.X.S., S.Z.L. and X.Y.L. wrote the manuscript. All authors read and commented on the manuscript.

## Additional information

**How to cite this article:** Lee, Y. H. *et al.* Nanoscale surface chemistry directs the tunable assembly of silver octahedra into three two-dimensional plasmonic superlattices. *Nat. Commun.* 6:6990 doi: 10.1038/ncomms7990 (2015).

## Supplementary Material

Supplementary Figures, Supplementary Tables, Supplementary Notes and Supplementary References. Supplementary Figures 1-17, Supplementary Tables 1-5, Supplementary Notes 1-3 and Supplementary References.

Supplementary Movie 1 Time-dependent changes in the interfacial positions and configurations of PVP-functionalized Ag octahedron.

Supplementary Movie 2 Time-dependent changes in the interfacial positions and configurations of C3SH-functionalized Ag octahedron.

Supplementary Movie 3 Time-dependent changes in the interfacial positions and configurations of C16SH-functionalized Ag octahedron.
