# Supplementary material for: Nanoscale surface chemistry directs the tunable assembly of silver octahedra into three two-dimensional plasmonic superlattices
Source: Nat Commun. 2015 Apr 29;6:6990. doi: 10.1038/ncomms7990 (PMC4421843; doi:10.1038/ncomms7990)
Supplement: Supplementary Figures, Supplementary Tables, Supplementary Notes and Supplementary References. — Supplementary Figures 1-17, Supplementary Tables 1-5, Supplementary Notes 1-3 and Supplementary References. [file ncomms7990-s1.pdf]

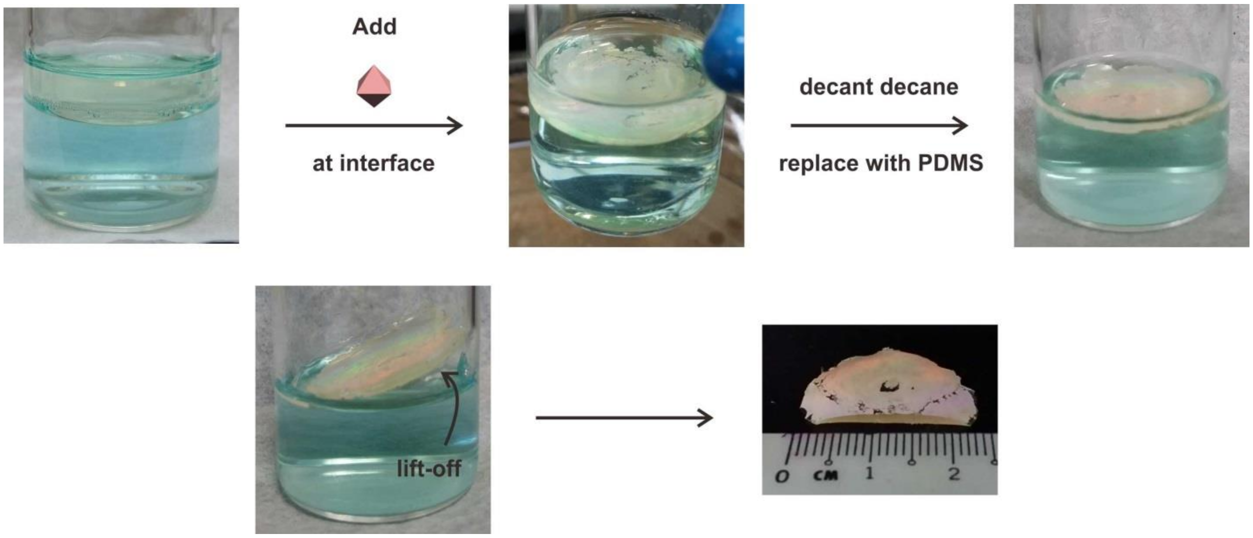

Supplementary Figure 1 | Digital images of GG experiments, corresponding to the respective stages shown in Fig. 1a. The aqueous phase is lightly dyed with methylene blue to aid the visualization of the originally transparent oil/water interface. The use of PDMS mold enables the octahedra superlattice to be removed from the physically cross-linked gel at the interface for subsequent characterization because chemical cross-linking of the PDMS networks occur during the PDMS polymerization process. Polymerization of PDMS occurs overnight at room temperature conditions.

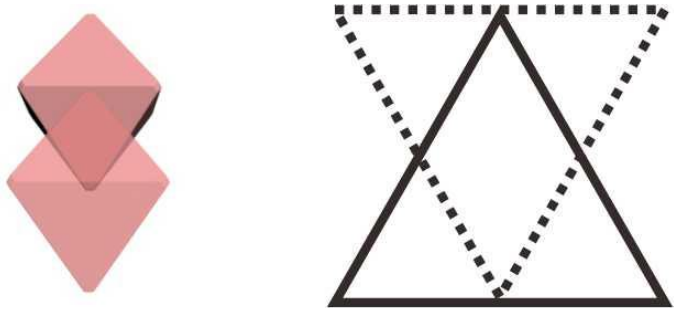

Supplementary Figure 2 | 2D projection of the contact areas between two neighboring Ag octahedra in the hexagonal close-packed arrangement. Each triangular facet of one octahedron can be divided into four smaller triangles, resulting in eight small triangles for two contacting octahedra. Hexagonal close-packing gives rise to a total of four small triangles overlapping each other, resulting in an overlap of 50 % in terms of the sum of eight small triangles (equivalent to two triangular facets of two neighboring octahedra).

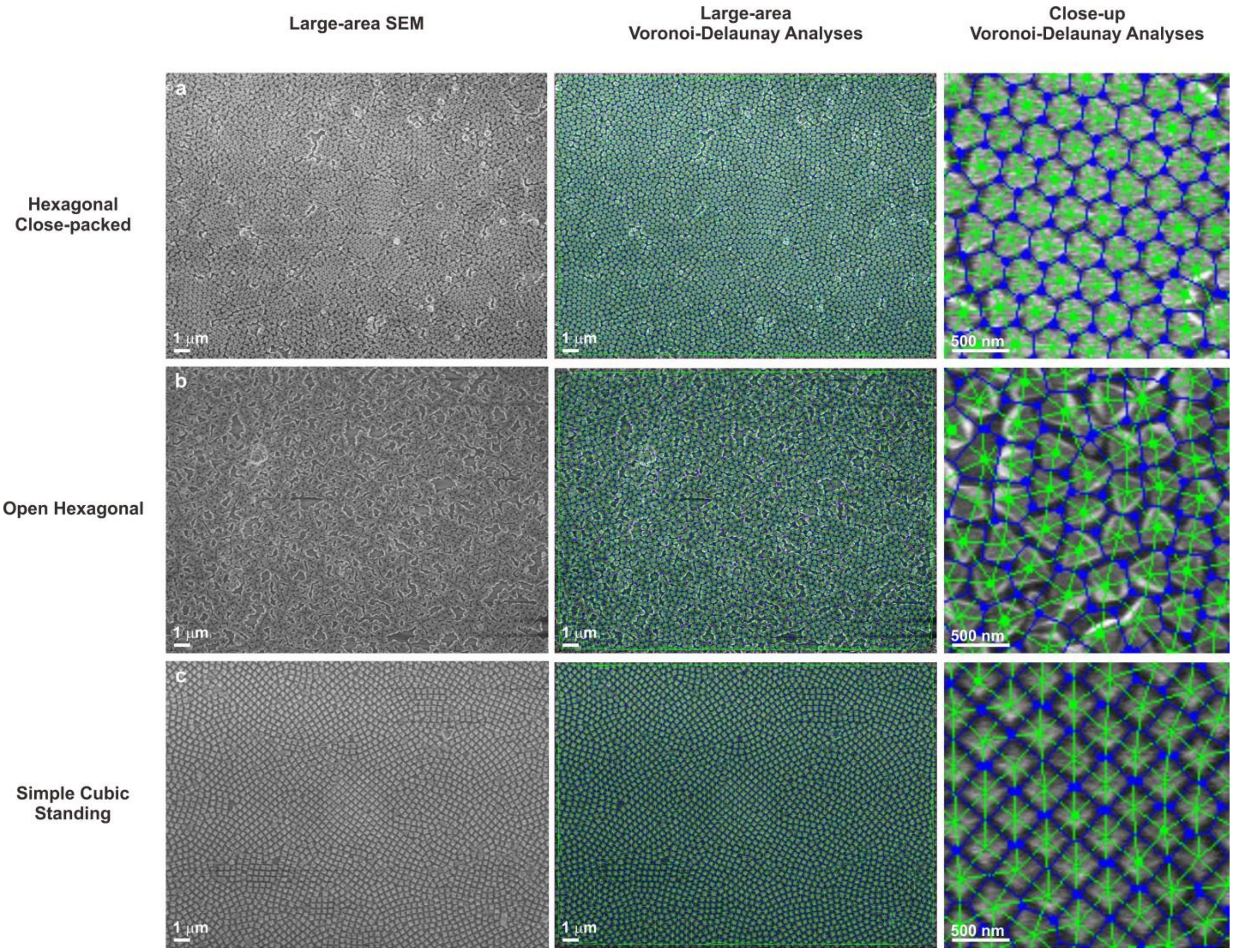

Supplementary Figure 3 | Order analyses of the superlattices. Voronoi-Delaunay cells for the hexagonal close-packed monolayer formed using PVP **(a)**, the open hexagonal monolayer formed using C3SH **(b)**, and the square lattice of standing octahedra formed using C16SH **(c)**. The three 2D superlattices of Ag octahedra exhibit long-range order. In addition to mapping the radial distribution functions (Fig. 1e, i, m), the Voronoi cells and Delaunay triangulation of the assembled 2D superlattices are also defined. Both the hexagonal close-packed and open hexagonal octahedra monolayer gives rise to a honeycomb network of hexagonal Voronoi cells (blue lines) whereas the Voronoi cells of the square lattice of standing octahedra corresponds to a network of squares (Fig. S6c). Complementary Delaunay triangulation is also observed for the three superlattices (green lines).

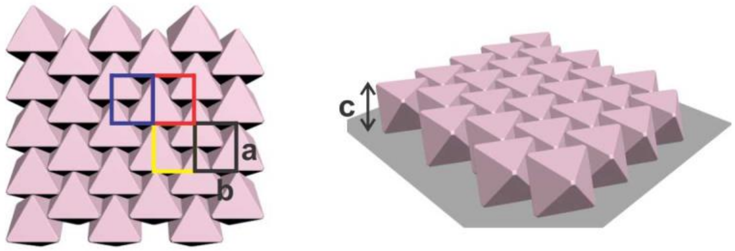

Supplementary Figure 4 | Determining the packing efficiency of hexagonal close-packed monolayer of Ag octahedra. The packing efficiency is calculated to be 89% (8/9). The simplest repeat unit of the hexagonal close-packed Ag octahedra is shown by the rectangles in (a). The repeat unit is filled with one octahedron, with the remaining space filled with four tetrahedral holes. Assuming the edge length of a single octahedron is l,

Length of a = $\frac{\sqrt{3}}{2}l$

Length of b = $\frac{3}{4}l$

Length of c = $\frac{\sqrt{6}}{3}l$

Volume of repeat unit = $a \times b \times c$

$= \frac{\sqrt{3}}{2}l \times \frac{3}{4}l \times \frac{\sqrt{6}}{3}l$

$= \frac{3\sqrt{2}}{8}l^3$

Volume of one octahedron = $\frac{\sqrt{2}}{3}l^3$

Packing efficiency of hexagonal close-packed array = $\frac{\frac{\sqrt{2}}{3}l^3}{\frac{3\sqrt{2}}{8}l^3}$

$= \frac{8}{9}$

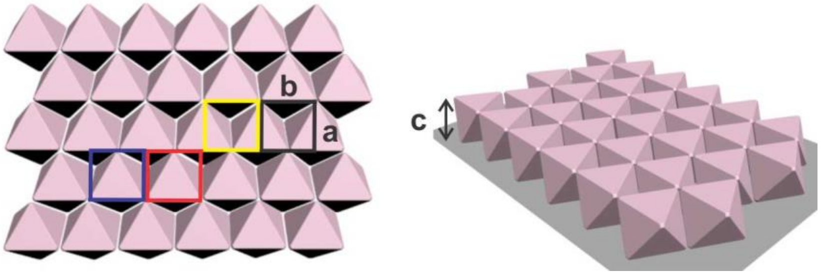

Supplementary Figure 5 | Determining the packing efficiency of open hexagonal monolayer of Ag octahedra. The packing efficiency is calculated to be 67% (2/3). The simplest repeat unit of the hexagonal open structure is shown by the rectangles in the figure above. The repeat unit is filled with one octahedron, with the remaining space filled with two tetrahedral holes.

Length of a = $\frac{\sqrt{3}}{2}l$

Length of b = l

Length of c = $\frac{\sqrt{6}}{3}l$

Volume of repeat unit $= a \times b \times c$

$$= \frac{\sqrt{3}}{2}l \times l \times \frac{\sqrt{6}}{3}l$$

$$= \frac{\sqrt{2}}{2}l^3$$

Volume of one octahedron $= \frac{\sqrt{2}}{3}l^3$

Packing efficiency of hexagonal close-packed array $= \frac{\frac{\sqrt{2}}{3}l^3}{\frac{\sqrt{2}}{2}l^3}$

$$= \frac{2}{3}$$

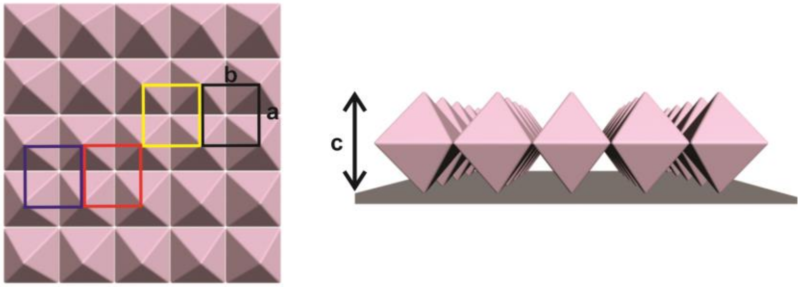

Supplementary Figure 6 | Determining the packing efficiency of square lattice of standing Ag octahedra. The packing efficiency is calculated to be 33% (1/3). The simplest repeat unit of the standing structure is shown by the rectangles in the figure above. The repeat unit is filled with one octahedron, with the remaining space filled with two tetrahedral holes.

Length of a = l

Length of b = l

Length of c = $\sqrt{2}l$

Volume of repeat unit $= a \times b \times c$

$= l \times l \times \sqrt{2}l$

$= \sqrt{2}l^3$

Volume of one octahedron $= \frac{\sqrt{2}}{3} l^3$

Packing efficiency of standing octahedra $= \frac{\frac{\sqrt{2}}{3}l^3}{\sqrt{2}l^3}$

$= \frac{1}{3}$

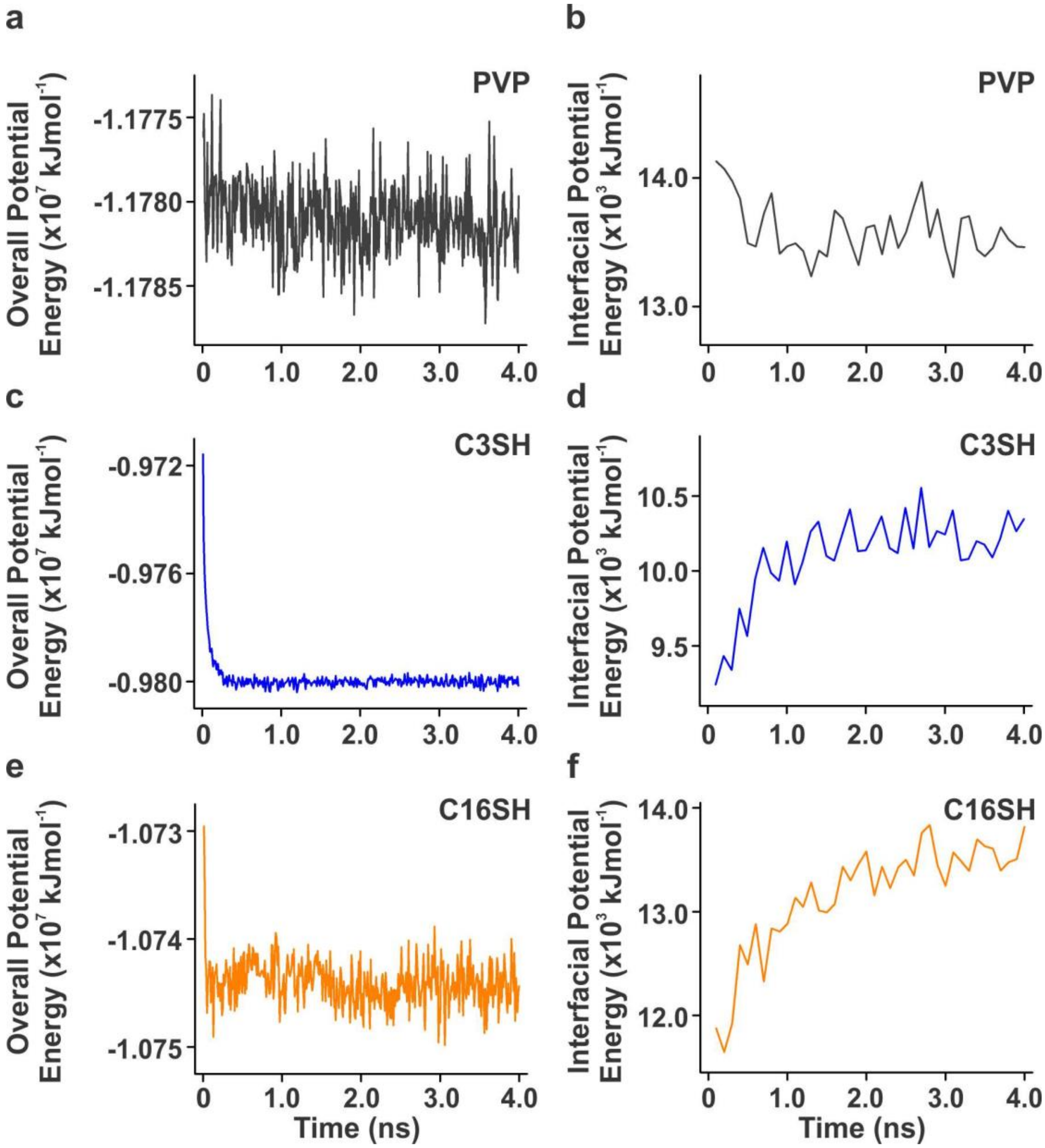

Supplementary Figure 7 | Overall potential energy and interfacial potential energy of the various simulated systems. The overall potential energy **(a,c,e)** in all cases decreases to reach a more thermodynamically stable state. A decrease in interfacial energy **(b,d,f)** is observed for PVP, while an increase is observed for C3SH and C16SH. This increase in interfacial energy arises from the particle moving into the oil phase completely.

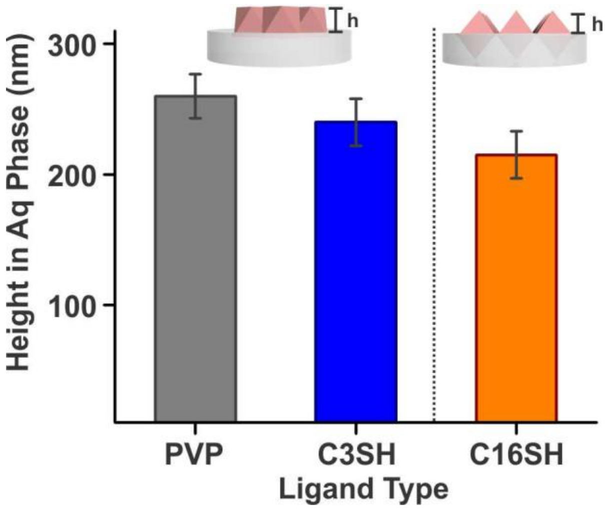

Supplementary Figure 8 | Topological characterization of the heights of variously functionalized Ag octahedra remaining in the aqueous phase. The height of the exposed Ag octahedra on the PDMS surface correspond to their heights and orientations in the aqueous phase because the PDMS mold itself replaces the oil phase during the lift-off process. PVP- and C3-octahedra are both planar with the triangular facet parallel to the oil/water interface; the heights of octahedra protrusion from the PDMS surface are (260 ± 17) nm and (240 ± 18) nm respectively. Since the distance between two parallel planes of the 356 nm Ag octahedron is 291 nm, ~ 31 nm (11 %) and 51 nm (18 %) of the PVP- and C3-octahedra are in contact with the oil phase respectively (Supplementary Table 1). The standing configuration for C16-octahedra is also evident in the AFM measurements, with the protrusion height of the Ag octahedra from the PDMS surface at around (215 ± 18) nm. Since the orthogonal distance between two tips of the 356 nm Ag octahedra is 503 nm, approximately 57 % of the Ag octahedra is in contact with the oil phase (Supplementary Table 4). Error bars correspond to standard deviation of the measurements collected from a minimum of 50 particles for each functionality.

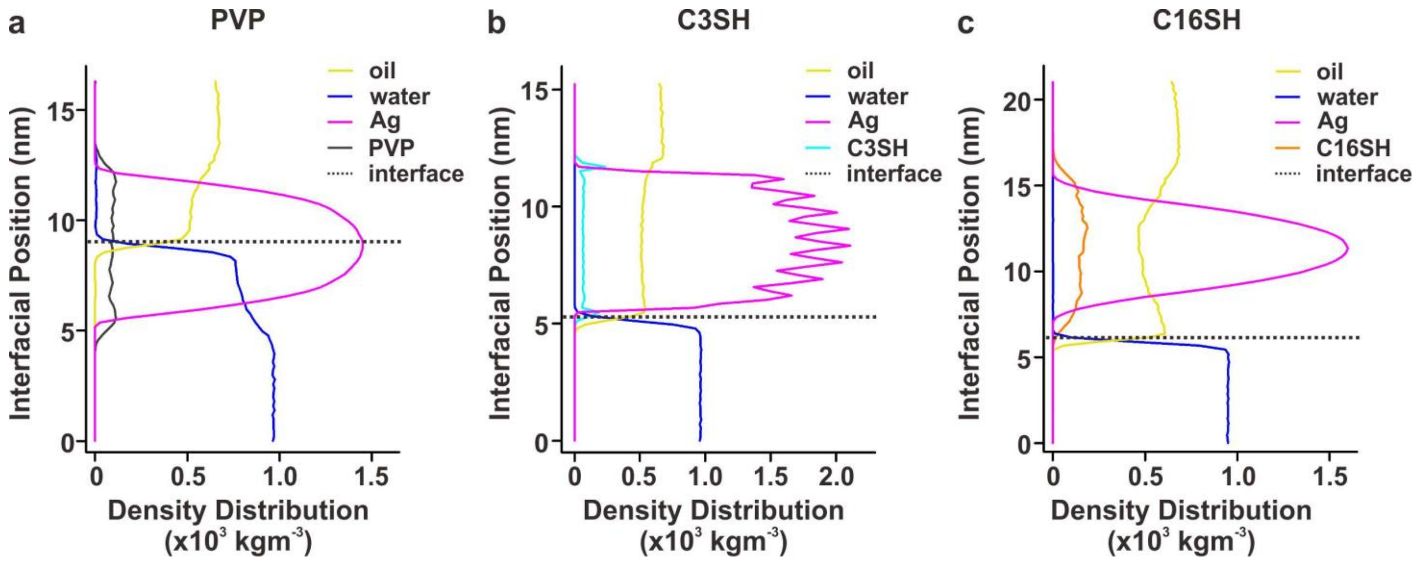

Supplementary Figure 9 | Determining the interfacial position of the Ag octahedra in the simulations. Density distribution profiles for PVP- **(a)**, C3SH- **(b)**, and C16SH-functionalized **(c)** Ag octahedron. The dash lines indicate the interface position, with the regions above corresponding to the oil phase, and the regions below corresponding to the aqueous phase. The density profiles of Ag, water and oil along the vertical direction of the simulation box are used to determine the interfacial positions of the variously functionalized Ag octahedron and to obtain the detailed potential energy profiles of the surface ligands. The interface between oil and water is defined by the decrease in the density of water to 10 %. The extent of Ag and ligand density profiles rising above this position is used to estimate the simulated height ratio of Ag octahedron in contact with the oil phase.

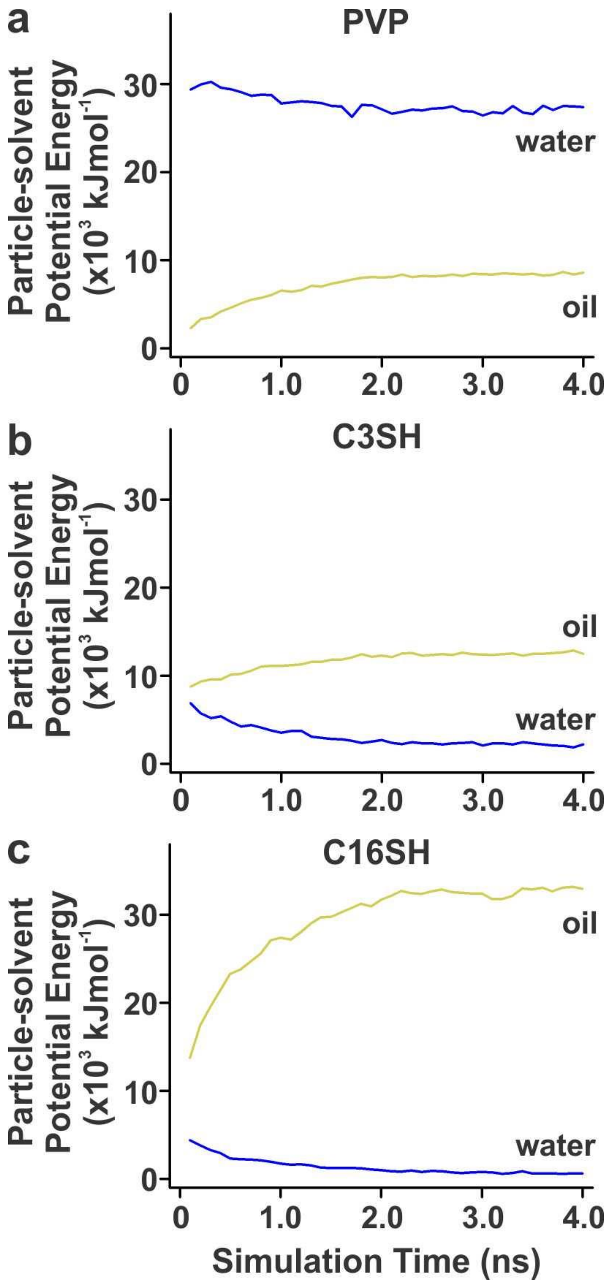

Supplementary Figure 10 | Changes in the interaction potential energy of the various ligands on Ag surface with both the aqueous and oil phases over time. A switch from stronger particle-water interaction to stronger particle-oil interaction is observed when the ligand used becomes increasingly hydrophobic.

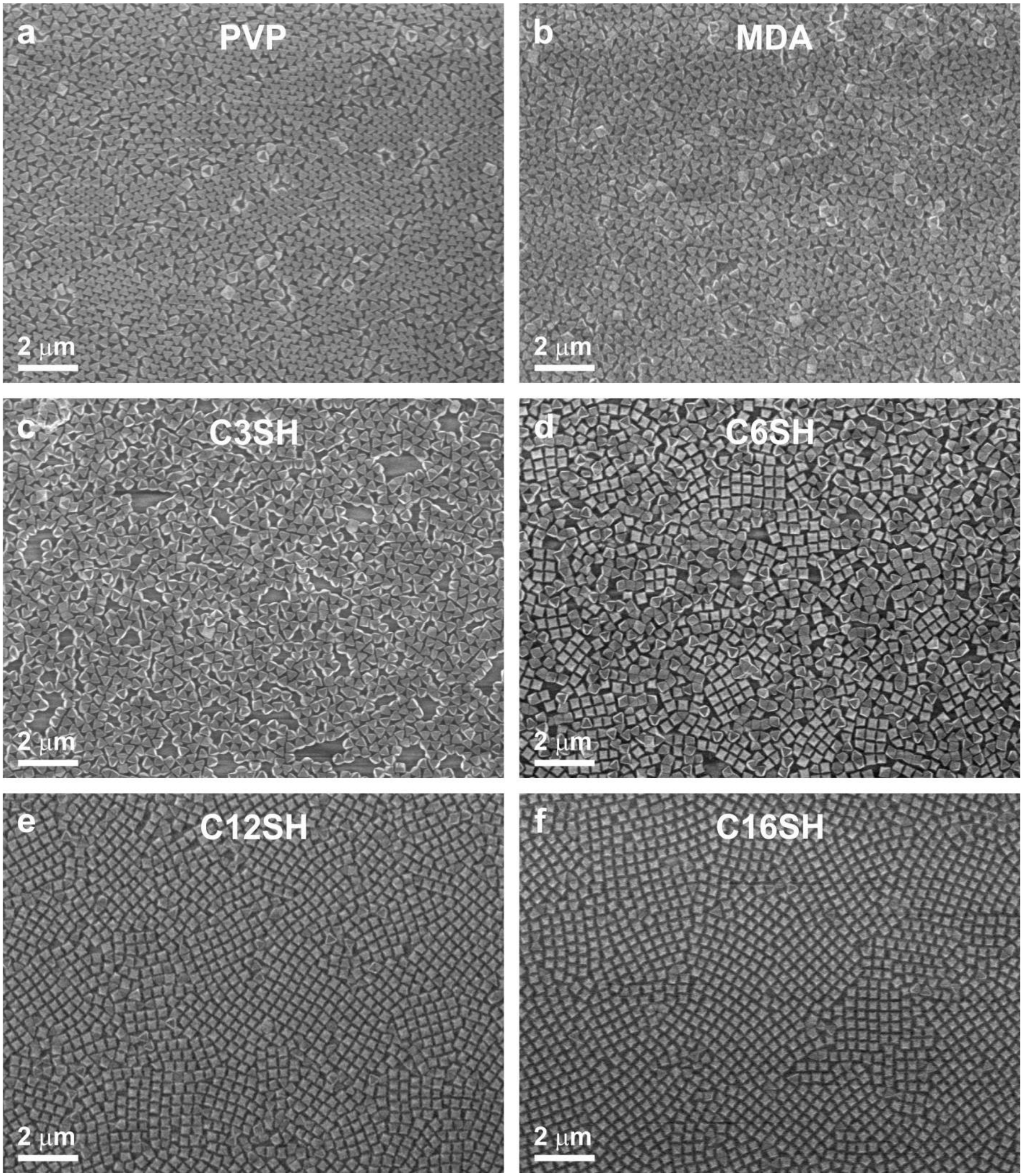

Supplementary Figure 11 | Change in the superlattice structure as the surface wettability of Ag octahedra used for the self-assembly becomes increasingly hydrophobic. Large-area SEM of the various self-assembled structures formed using PVP- **(a)**, MDA- **(b)**, C3SH- **(c)**, C6SH- **(d)**, C12SH- **(e)**, and C16SH-functionalized **(f)** Ag octahedra. The bulk contact angles measured on a thiol-functionalized Ag film are $(39 \pm 3)^o$, $(69 \pm 5)^o$, $(83 \pm 2)^o$, $(95 \pm 3)^o$, $(101 \pm 4)^o$, $(110 \pm 2)^o$, respectively.

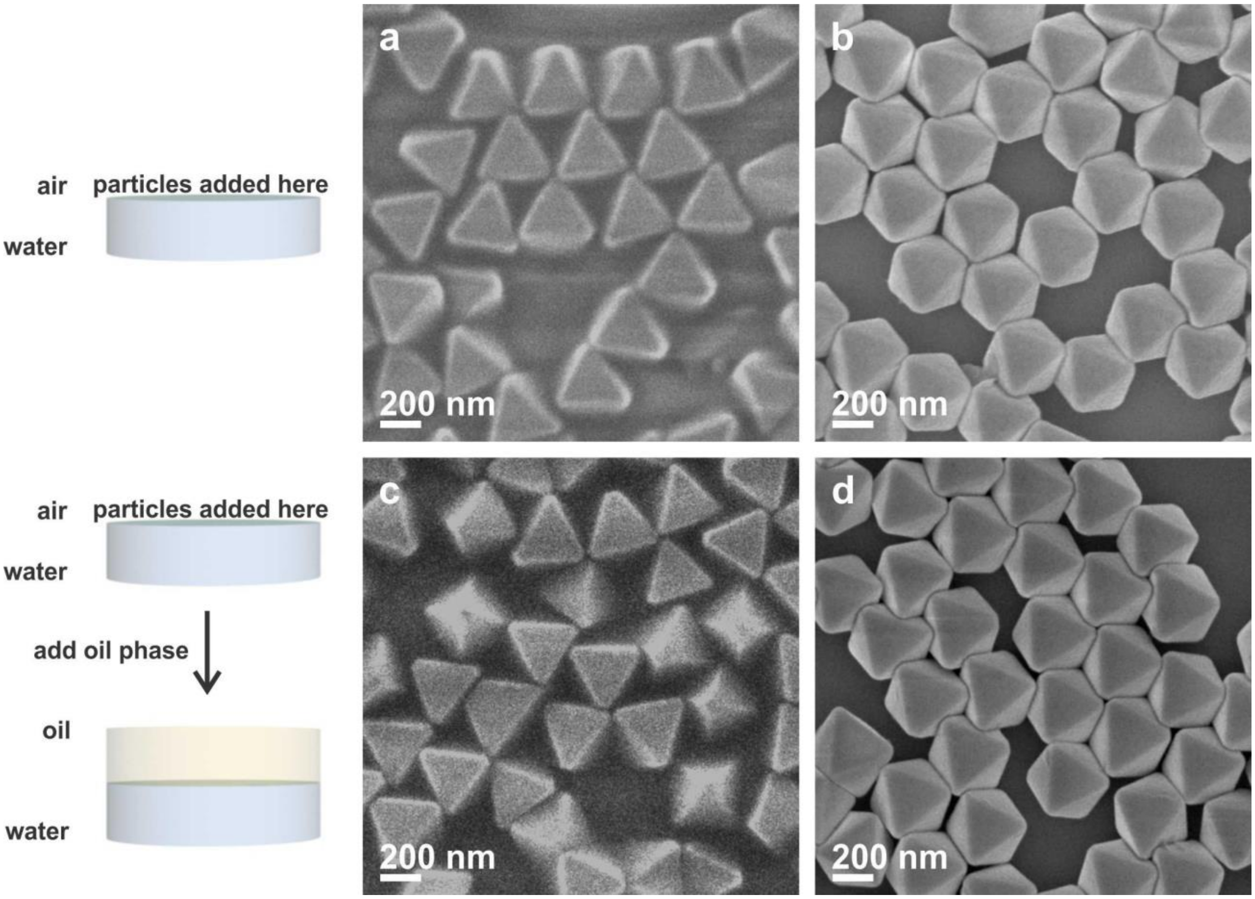

Supplementary Figure 12 | Importance of liquid-liquid interfaces in achieving standing configuration. C16SH-functionalized octahedra added at the air/water interface resulted in open hexagonal structure instead of square standing array in both the gel-trapping experiments **(a)** and dip-up self-assembly technique **(b)**. The low hydrophilic/hydrophobic potential ratio of C16-octahedra implies that they are unable to remain buoyant standing in air in the absence of the oil phase. **c,d,** The open hexagonal structure remains fixed even when the oil phase is added after the addition of the particles in both cases. There is no energetic incentive to vary the superlattice structure upon the addition of an organic phase since the air/water interfacial energy is much higher than that of the oil/water.

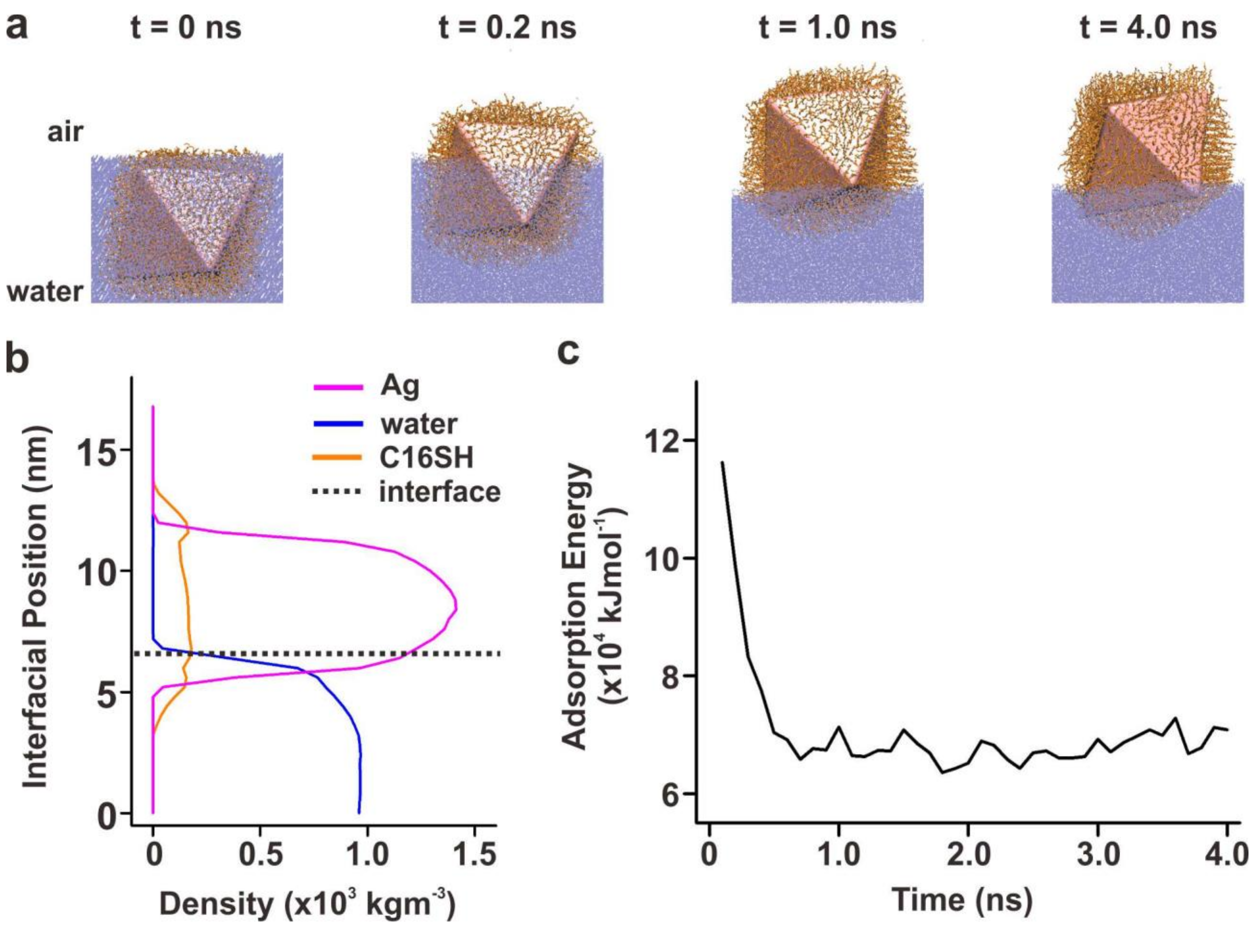

Supplementary Figure 13 | Molecular dynamics simulations of the interfacial behavior of C16SH-functionalized Ag octahedron at the air/water interface. a,b, Ag octahedron moves across the air/water interface over time. **c,** The particle-water potential energy remains relatively low due to the hydrophobicity of the C16-octahedron.

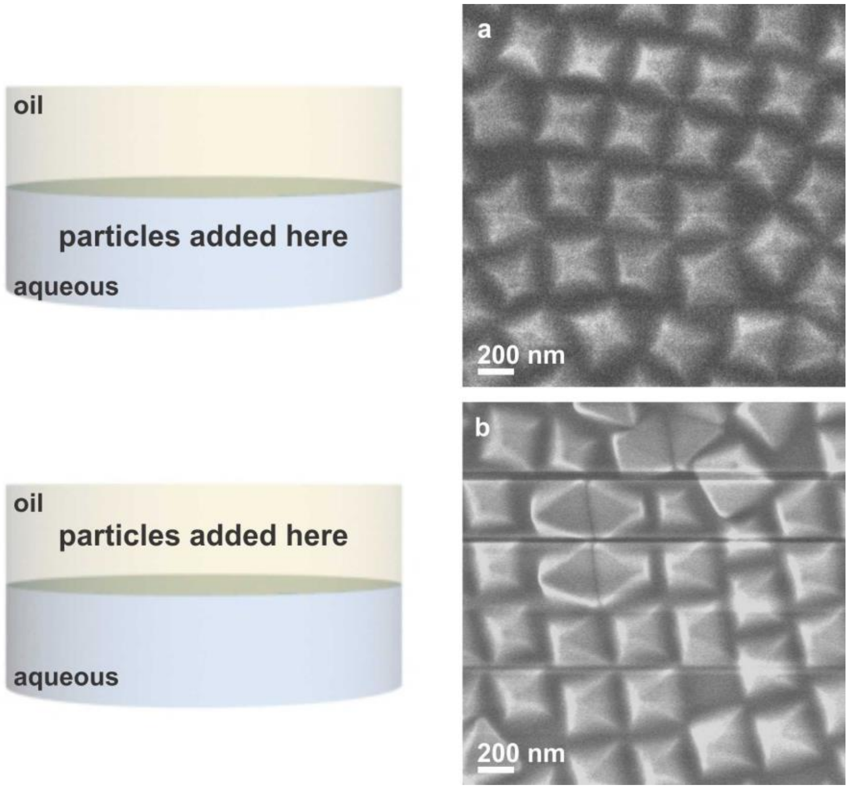

Supplementary Figure 14 | Investigating the influence of particle introduction location on the formation of the square superlattice. The locations at which the octahedra are added to the oil/water interface does not matter, since the addition from both the aqueous **(a)** and oil phase **(b)** gives rise to standing octahedra for the gel-trapping self-assembly experiments. Particles move to the interface spontaneously to achieve thermodynamic equilibrium and to minimize unfavorable contact between the two immiscible phases, verified through simulations.

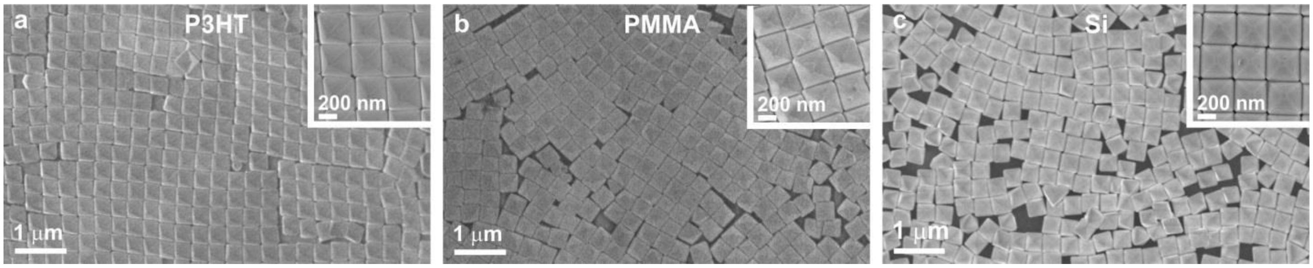

Supplementary Figure 15 | Demonstrating the versatility of assembling the superlattices on various platforms. Transferring the standing Ag octahedra to P3HT **(a)**, PMMA **(b)**, and Si substrates **(c)** can be achieved without significant structural distortion. Superlattice transfer to polymeric substrates is similar to method used for transfer to PDMS, except that P3HT and PMMA solutions are introduced over the hardened gel instead. The ease of assembly of the 2D Ag octahedra superlattices on different polymeric platforms opens up new opportunities in 'flexible plasmonics' and potentially even 'flexible photovoltaics'.

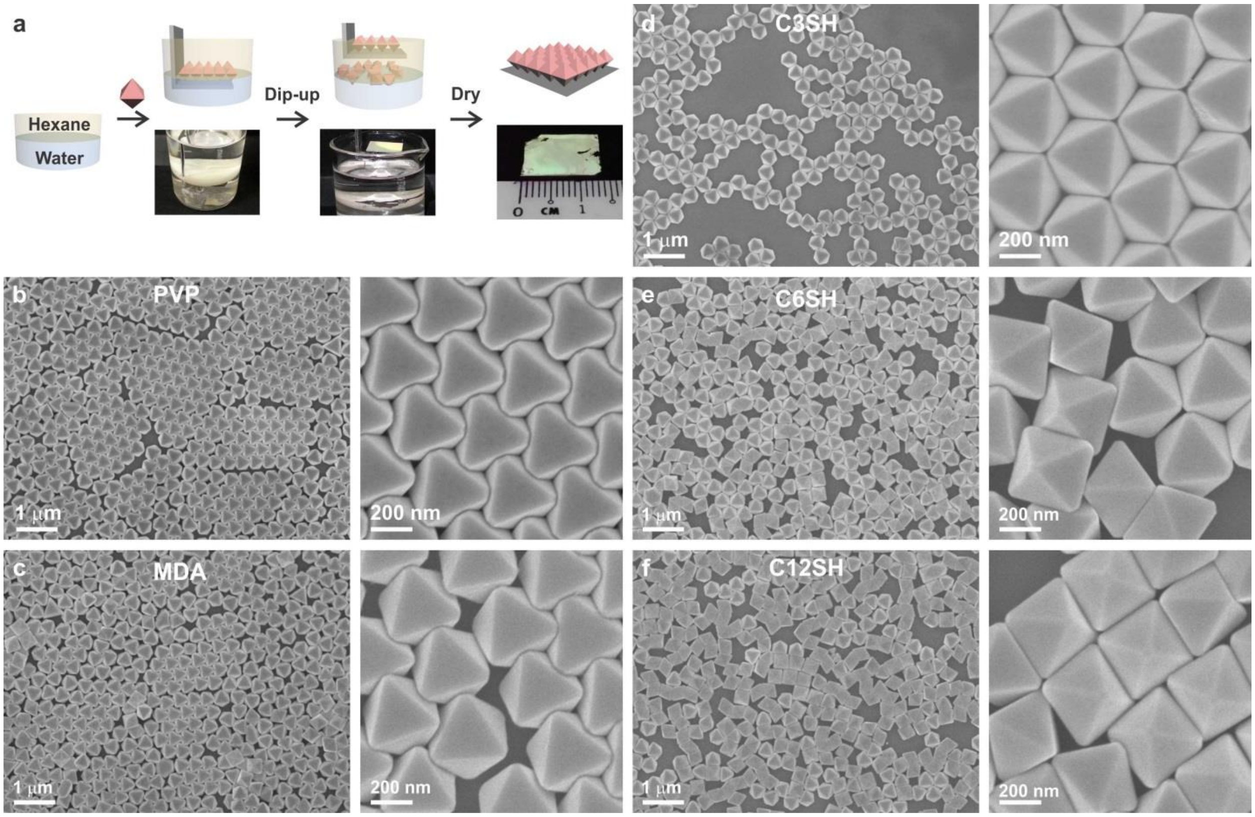

Supplementary Figure 16 | Transferring the superlattices onto a Si substrate using a dip-up self-assembly scheme. a, Schematic illustration of the dip-up self-assembly of Ag octahedra for transferring the monolayers onto a Si substrate. SEM images of monolayers formed using the dip-up self-assembly for PVP- **(b)**, MDA- **(c)**, C3SH- **(d)**, C6SH- **(e)**, and C12SH-functionalized **(f)** Ag octahedra. The relative yields of the Ag octahedra monolayers on Si substrates are lower, likely caused by the disruption of the oil/water interface during the dip-up process, causing the octahedra arrays to lose their long-range order.

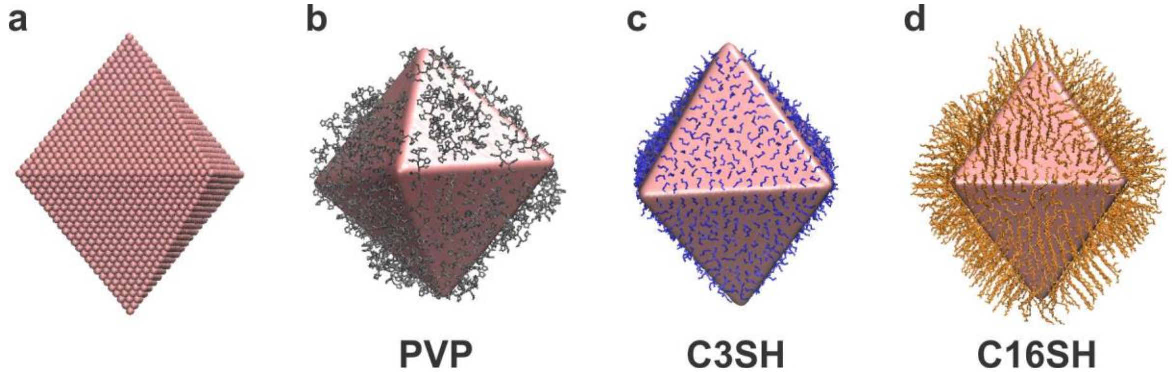

Supplementary Figure 17 | Octahedron models constructed for simulations. a, Bare Ag octahedron built using 10425 Ag atoms; **b,c,d,** Ag octahedron functionalized with PVP **(b)**, C3SH **(c)**, and C16SH **(d)**.

Supplementary Table 1 | AFM measurements and estimated single-particle contact angles of Ag octahedra functionalized with different ligands.

Array	AFM Height (aq. phase) / nm	Height in Oil / nm	% Immersed in Oil	% Immersed in Oil (Simulation)	Hydrophobic/Hydrophilic Ratio (Simulation)
Hexagonal Close-packed	260	31	11	49	0.3
Open Hexagonal	240	51	18	100	6
Square Standing	215	288	57	100	52

Supplementary Table 2 | Vibrational mode assignments of the SERS bands observed from 4-MBT at an excitation wavelength of 532 nm.

SERS Band / cm^{-1}	Vibrational Modes
1080	combination of phenyl ring-breathing, C-H in-plane bending, and C-S stretching
1600	phenyl stretching

Supplementary Table 3 | Parameters for non-bonded and bonded interactions of PVP.

Atom	Mass	$C_i^{(6)}$ (kJ×mol^{-1}nm^{6})	$C_i^{(12)}$ (kJ×mol^{-1}nm^{12})
CH3	15.0350	0.0096138025	2.6646244e-05
CH2	14.0270	0.0074684164	3.3965584e-05
CH1	13.0190	0.00606841	9.70225e-05
O	15.9994	0.0022619536	1e-06
NR	14.0067	0.0024364096	3.389281e-06
C	12.0110	0.0023406244	4.937284e-06

Bond		
	b_0(nm)	K_b(kJ.mol^{-1}nm^{-2})
CH3- CH1	0.153	7150000.0
CH2- CH1	0.153	7150000.0
N- CH2	0.148	5730000.0
N- CH1	0.148	5730000.0
C-N	0.133	11800000.0
C- O	0.123	16600000.0
C-CH2	0.139	8660000.0

Angle		
	θ_0(deg)	K_θ(kJ.mol^{-1}rad^{2})
O-C-CH2	121.0	685.0
O-C-NR	120.0	560.0
CH2-C-NR	108.0	465.0
C-CH2-CH1	104.0	444.4
C-NR-CH1	125.0	375.0
NR-CH1-CH3	111.0	530.0

Supplementary Table 4 | Parameters for non-bonded and bonded interactions of the C16SH and C3SH.

Atom	Mass	$C_i^{(6)}$ (kJ×mol^{-1}nm^{6})	$C_i^{(12)}$ (kJ×mol^{-1}nm^{12})
CH3	15.0350	0.0096138025	2.6646244e-05
CH2	14.0270	0.0074684164	3.3965584e-05
S	32.0600	0.0099840064	1.3075456e-05
H	1.0080	0	0

Bond		
	b_0(nm)	K_b(kJ.mol^{-1}nm^{-2})
CH3- CH2	0.153	7150000.0
CH2- CH2	0.153	7150000.0
S- H	0.133	8870000.0
S- CH2	0.183	5620000.0

Angle		
	θ_0(deg)	K_θ(kJ.mol^{-1}rad^{2})
H-S-CH2	96.0	405.0
S-CH2-CH2	113.0	545.0
CH2-CH2-CH2	109.5	520.0
CH2-CH2-CH3	109.5	520.0

Supplementary Table 5 | Parameters for non-bonded and bonded interactions of the hexane.

Atom	Mass	$C_i^{(6)}$ (kJ×mol^{-1}nm^{6})	$C_i^{(12)}$ (kJ×mol^{-1}nm^{12})
CH3	15.0350	0.0096138025	2.6646244e-05
CH2	14.0270	0.0074684164	3.3965584e-05

Bond		
	b_0(nm)	K_b(kJ.mol^{-1}nm^{-2})
CH3- CH2	0.153	7150000.0
CH2- CH2	0.153	7150000.0

Angle		
	θ_0(deg)	K_θ(kJ.mol^{-1}rad^{2})
CH2-CH2-CH2	109.5	520.0
CH2-CH2-CH3	109.5	520.0

Supplementary Note 1: Molecular Dynamics Simulations

The aim of utilizing molecular dynamics simulations is to seek a fundamental understanding on the interfacial behavior of a single anisotropic nanoparticle at an oil/water interface. We make use of the simulations to gain insights on how changes to the surface wettability of Ag octahedron arising from the use of various ligands (PVP, C3SH, C16SH) leads to structural changes observed in the self-assembly experiments. For this purpose, we employ an all-atomic molecular dynamics simulation model rather than a coarse-grain model to focus on the surface interactions occurring on the nanoparticle surface with the solvents. The thiol molecules are assumed to form a self-assembled monolayer on the octahedron surface, with experimental density of ~4.5×10^{14} molecules/cm^{2}[, 1]; we also assume monolayer coverage for PVP, with the PVP chain non-specifically adsorbed on the octahedron surface.

Computational details. The GROMACS 4.07 simulation package[2] and GROMOS96 force field[3] were used for all our MD simulations. Two neighboring atoms interact with each other through van der Waals interactions, which is treated using a 12-6 Lennard - Jones (LJ) potential summed over all pairs of atoms i and j. The LJ potential may also be written in the following form:

$$V_{LJ}(\mathrm{r_{ij}}) = 4\varepsilon_{ij}((\frac{\sigma_{ij}}{r_{ij}})^{12} - (\frac{\sigma_{ij}}{r_{ij}})^{6}) \tag{1}$$

where r_{ij} is the distance between the interacting pairs of atoms, σ_{ij} and ε_{ij} are the LJ parameters between atoms. The GROMACS LJ potential parameters $C_i^{(6)}$ and $C_i^{(12)}$ can be defined using the combination rules:

$$C_i^{(6)} = 4\varepsilon_i \sigma_i^{6} \tag{2}$$

$$C_i^{(12)} = 4\varepsilon_i \sigma_i^{12} \tag{3}$$

The combinations for different atom-types can be computed according to the combination rule:

$$C_{ij}^{(6)} = (C_i^{(6)} C_j^{(6)})^{\frac{1}{2}} \tag{4}$$

$$C_{ij}^{(12)} = (C_i^{(12)} C_j^{(12)})^{\frac{1}{2}} \tag{5}$$

To investigate the configuration evolution of Ag octahedron with various ligands at the oil/water interface, the LJ parameters (σ_0 = 0.2955 nm and ε_0 = 19.0790 kJ/mol) for Ag atoms were used[4]. The oil phase (hexane or decane), poly(vinylpyrrolidone) (PVP), 1-propanethiol (C3SH), and 1-hexadecanethiol (C16SH) molecular models employed in this study were generated from the small-molecule topology generator PRODRG and the $C^{(6)}$ and $C^{(12)}$ parameters are listed in Supplementary Tables 3, 4, and 5. The water phase was modeled using the single point charge (SPC) model, with the bond lengths and angles held constant through the use of the SETTLE algorithm. Bond lengths of molecules were constrained using the

LINCS algorithm. The cutoff distance for short-range non-bonded interactions was chosen to be 12 Å and long-range electrostatic forces were computed using the reaction-field approach[5,6].

The Ag octahedron used in the simulation was constructed by an all-atomic model using 10425 Ag atoms, as shown in Fig. S17a, corresponding to an edge length of ~ 7.5 nm. 129 PVP chains each built with 8 repeat units are randomly adsorbed onto the surface Ag atoms, as shown in Fig. S17b; 768 thiol molecules (C3SH and C16SH) were chemisorbed onto the Ag atoms to form a self-assembled monolayer, as shown in Fig. S17c and d. Simulations were run over 4 ns with steps of 2 fs, over the course of which the potential energies of the systems became stable. The above described setups demanded 53–66 24-core CPU hours on 2.13 GHz Intel Xeon Nehalam processors per simulation. As such, the size of the Ag octahedron was fixed at 7.5 nm to alleviate the computational demands while retaining the ability to focus on the events occurring at the surface of the Ag octahedron at the oil/water interface.

The simulations were started from the preassembled system consisting of two abutting thick slabs of water and hexane, with various ligand-functionalized Ag octahedron immersed in the aqueous phase. The water slab was composed of 11955 water molecules, and the oil slab was composed of 2341 hexane molecules. The dimensions of the simulation box were $16 \times 16 \times 24$ nm^3. Simulations were performed using the NPT ensemble. The temperature was maintained at 300 K using the Berendsen temperature coupling method and Berendsen bath coupling scheme was used to keep a constant normal pressure of 1 bar[7].

The last 400 ps trajectory was used for analysis to derive the density distribution profile of the four components (Ag for Ag octahedron, surface ligands, water, and oil) during which the potential energy, the dimensions of the simulation box remained stable. The density profiles of the components aforementioned (Ag, water and oil) along the vertical direction of the simulation box were used to determine the interfacial positions of the variously functionalized Ag octahedron and to obtain the detailed potential energy profiles of the surface ligands. The interface between oil and water was defined by the decrease in the density of water to 10 %. The extent of Ag and ligand density profiles rising above this position was used to estimate the simulated height ratio of Ag octahedron in contact with the oil phase. In addition, snapshot pictures at various time intervals during the simulation were prepared using VMD to show the interfacial configuration of the Ag octahedron[8].

Supplementary Note 2: Overall Potential Energy and Interfacial Energy Changes

Thermodynamic stability drives the variously functionalized Ag octahedron towards the oil/water interface over the course of the all-atomic molecular dynamics simulations. The total potential energies of all the systems are lower at the end of the simulation than at the beginning, reaching constant values after the simulation (Supplementary Fig. 7). In conjunction with the movement of the Ag octahedron in the three cases to the oil/water interface (Fig. 2), the decrease in overall potential energies indicates that this movement is a spontaneous process. The Ag octahedron breaches the oil/water interface and subsequent deforms the interface to minimize the energetically unfavorable contact between the immiscible oil and aqueous phases [9,10]. Consequently, the Ag octahedron is trapped at the oil/water interface.

In addition, the interfacial potential energies ($E_{interfacial}$) of the systems were derived using the following relationship:

$$E_{interfacial} = (E_{water} + E_{oil} + E_{Ag+ligand}) - E_{system} \quad (6)$$

where E_{water} is the total potential energy of the aqueous phase; E_{oil} is the total potential energy of the oil phase; $E_{Ag+ligand}$ is the total potential energy of the ligand and Ag core; E_{system} is the total potential of the entire system.

The interaction of the octahedron with the aqueous ($E_{water-(Ag+ligand)}$) and oil phases ($E_{oil-(Ag+ligand)}$) are derived from the following equations:

$$E_{water-(Ag+ligand)} = (E_{water} + E_{(Ag+ligand)}) - E_{water+Ag+ligand} \quad (7)$$

$$E_{oil-(Ag+ligand)} = (E_{oil} + E_{(Ag+ligand)}) - E_{oil+Ag+ligand} \quad (8)$$

where E_{water} is the total potential energy of the aqueous phase, E_{oil} is the total potential energy of the oil phase, $E_{Ag+ligand}$ is the total potential energy of the ligand and Ag core, $E_{water+Ag+ligand}$ is the total potential energy of aqueous phase and the ligand and Ag core, $E_{oil+Ag+ligand}$ is the total potential energy of oil phase and the ligand and Ag core.

Supplementary Note 3: Calculating SERS Enhancement Factors

$$\text{SERS EF} = \frac{I_{SERS}}{N_{SERS}} \div \frac{I_{ref}}{N_{ref}} \quad (9)$$

$I_{surface}$ (hexagonal close-packed) = 7.30 cps
$I_{surface}$ (open hexagonal) = 5.95 cps
$I_{surface}$ (cubic standing) = 16.80 cps
$I_{solution}$ = 0.274 cps (1 M 4-MBT in ethanol)

In solution

$N_{solution}$ = $V_{solution} \times C_{4\text{-MBT}} \times$ Avogadro's number
$= \pi \times \frac{x}{2} \times \frac{y}{2} \times z \times c \times Avogadro's\ number$
$N_{solution}$ = 1.26×10^9 for 1 M 4-MBT in ethanol

Laser resolution in ethanol
x = 910 nm
y = 680 nm
z = 4320 nm
c = 1000 mol/m^3

Laser resolution in air:
x = 520 nm
y = 380 nm
z = 810 nm

Area of laser spot $= \pi \times \frac{x}{2} \times \frac{y}{2}$
$= 1.552 \times 10^5\ nm^2$
$= 1.552 \times 10^{-1}\ \mu m^2$

Octahedra density estimated from counting the number of octahedra over areas of 320 μm^2 for each array:

D (hexagonal close-packed) = 8.8 octahedra/μm^2
D (open hexagonal) = 7.4 octahedra/μm^2
D (square standing) = 7.1 octahedra/μm^2

Number of Ag octahedra within the laser spot:
N (hexagonal close-packed) = 1.360
N (open hexagonal) = 1.141
N (square standing) = 1.109

Exposed surface area of one Ag octahedron in three arrays:
S (hexagonal close-packed) = 4.274×10^5 nm^2
S (open hexagonal) = 3.915×10^5 nm^2
S (square standing) = 1.604×10^5 nm^2

Exposed surface area of Ag octahedra within the laser spot:
S (hexagonal close-packed) = 5.811×10^5 nm^2
S (open hexagonal) = 4.468×10^5 nm^2
S (square standing) = 1.779×10^5 nm^2

Taking 4.5×10^{14} molecules/cm^2 for a monolayer of 4-MBT on silver [1], number of 4-MBT molecules adsorbed on the particles surface within the laser spot:

N (hexagonal close-packed) = 4.5 molecules/$nm^2 \times 5.811 \times 10^5$ nm^2
= 2.614×10^6

N (open hexagonal) = 4.5 molecules/$nm^2 \times 4.468 \times 10^5$ nm^2
= 2.010×10^6

N (cubic standing) = 4.5 molecules /$nm^2 \times 1.779 \times 10^5$ nm^2
= 8.006×10^5

EF (hexagonal close-packed) = $\frac{7.30}{2.614\times10^6} \div \frac{0.274}{1.26\times10^9}$
= $\underline{1.3 \times 10^4}$

EF (open hexagonal) = $\frac{5.95}{2.010\times10^6} \div \frac{0.274}{1.26\times10^9}$
= $\underline{1.4 \times 10^4}$

EF (cubic standing) = $\frac{16.80}{8.006\times10^5} \div \frac{0.274}{1.26\times10^9}$
= $\underline{9.9 \times 10^4}$

Supplementary References

1 Love, J. C., Estroff, L. A., Kriebel, J. K., Nuzzo, R. G. & Whitesides, G. M. Self-assembled monolayers of thiolates on metals as a form of nanotechnology. *Chem. Rev.* **105**, 1103-1169 (2005).

2 Becraft, K. A., Moore, F. G. & Richmond, G. L. Charge reversal behavior at the CaF_2/H_2O/SDS interface as studied by vibrational sum frequency spectroscopy. *J. Phys. Chem. B* **107**, 3675-3678 (2003).

3 Van der Spoel, D. *et al.* GROMACS: fast, flexible, and free. *J. Comp. Chem.* **26**, 1701-1718 (2005).

4 Heinz, H., Vaia, R. A., Farmer, B. L. & Naik, R. R. Accurate simulation of surfaces and interfaces of face-centered cubic metals using 12-6 and 9-6 Lennard-Jones potentials. *J. Phys. Chem. C* **112**, 17281-17290 (2008).

5 Darden, T., York, D. & Pedersen, L. Particle mesh ewald: an Nlog(N) method for ewald sums in large systems. *J. Chem. Phys.* **98**, 10089-10092 (1993).

6 Essmann, U. *et al.* A smooth particle mesh ewald method. *J. Chem. Phys.* **103**, 8577-8593 (1995).

7 Berendsen, H. J. C., Postma, J. P. M., Vangunsteren, W. F., Dinola, A. & Haak, J. R. Molecular dynamics with coupling to an external bath. *J. Chem. Phys.* **81**, 3684-3690 (1984).

8 Humphrey, W., Dalke, A. & Schulten, K. VMD: Visual Molecular Dynamics. *J. Mol. Graph.* **14**, 33-38 (1996).

9 Cavallaro, M., Jr., Botto, L., Lewandowski, E. P., Wang, M. & Stebe, K. J. Curvature-driven capillary migration and assembly of rod-like particles. *Proc. Natl. Acad. Sci. U.S.A.* **108**, 20923-20928 (2011).

10 Furst, E. M. Directing colloidal assembly at fluid interfaces. *Proc. Natl. Acad. Sci. U.S.A.* **108**, 20853-20854 (2011).
